# A deep learning model for drug screening and evaluation in bladder cancer organoids

**DOI:** 10.3389/fonc.2023.1064548

**Published:** 2023-04-24

**Authors:** Shudi Zhang, Lu Li, Pengfei Yu, Chunyue Wu, Xiaowen Wang, Meng Liu, Shuangsheng Deng, Chunming Guo, Ruirong Tan

**Affiliations:** ^1^ School of Information Science and Engineering, Yunnan University, Kunming, China; ^2^ College of Life Sciences, Yunnan University, Kunming, China; ^3^ Center for Organoids and Translational Pharmacology, Translational Chinese Medicine Key Laboratory of Sichuan Province, Sichuan Institute for Translational Chinese Medicine, Sichuan Academy of Chinese Medicine Sciences, Chengdu, China

**Keywords:** deep learning, bladder cancer organoids, image segmentation, drug screening, U2Net model

## Abstract

Three-dimensional cell tissue culture, which produces biological structures termed organoids, has rapidly promoted the progress of biological research, including basic research, drug discovery, and regenerative medicine. However, due to the lack of algorithms and software, analysis of organoid growth is labor intensive and time-consuming. Currently it requires individual measurements using software such as ImageJ, leading to low screening efficiency when used for a high throughput screen. To solve this problem, we developed a bladder cancer organoid culture system, generated microscopic images, and developed a novel automatic image segmentation model, AU2Net (Attention and Cross U2Net). Using a dataset of two hundred images from growing organoids (day1 to day 7) and organoids with or without drug treatment, our model applies deep learning technology for image segmentation. To further improve the accuracy of model prediction, a variety of methods are integrated to improve the model’s specificity, including adding Grouping Cross Merge (GCM) modules at the model’s jump joints to strengthen the model’s feature information. After feature information acquisition, a residual attentional gate (RAG) is added to suppress unnecessary feature propagation and improve the precision of organoids segmentation by establishing rich context-dependent models for local features. Experimental results show that each optimization scheme can significantly improve model performance. The sensitivity, specificity, and F1-Score of the ACU2Net model reached 94.81%, 88.50%, and 91.54% respectively, which exceed those of U-Net, Attention U-Net, and other available network models. Together, this novel ACU2Net model can provide more accurate segmentation results from organoid images and can improve the efficiency of drug screening evaluation using organoids.

## 1 Introduction

Bladder cancer is a malignant tumour that occurs on the bladder mucosa and is a common malignancy of the urinary system. In recent years, the incidence of bladder cancer has risen rapidly, especially in men and the elderly population. According to the latest data from GLOBOCAN, bladder cancer ranks 13th in the ranking of malignant tumors, and its incidence rate ranks among the top 10 (1). Most patients with early bladder cancer are unaware of their condition and have no apparent symptoms, resulting in a low early diagnosis rate (2). Therefore, early detection and diagnosis are crucial for bladder cancer management.

An organoid (3–5) is a multicellular *in vitro* tissue cultured in three dimensions (3D). It can mimic the original tissue environment and is similar to the primary organ in terms of physical and chemical properties. Compared with cell lines and animal experiments currently and commonly conducted, organoid experiments have a broader application prospect in various preclinical and clinical studies. Human bladder cancer patients’ organoids had been established and exome sequencing confirmed that the spectrum of genetic mutation from cultured organoids is heterogeneous and may represent bladder cancer tumour evolution *in vivo* (6), in addition, patient derived organoids recapitulate the histopathological diversity of human bladder cancer. Therefore, bladder organoids could be used for drug screening and could serve as a preclinical platform for precision medicine. Indeed, the drug response using patient derived tumor organoids show partial correlations with mutational profiles and changes associated with drug resistance, especially the response can be validated in xenografts model (7), furthermore, the drug screen could be easily performed *in vitro* rapidly to match cancer patient’s unique mutation. Beside bladder cancer, 3D cell culture and organoid approaches are being increasingly used for other cancer types and even broader scenario in basic research and drug discovery in a variety of diseases (8). Recent advances in these technologies, which enable the *in vitro* study of these tissue-like structures, are very important for assessment of heterogenous diseases, such as colorectal cancer (9). As well, the use of 3D culture will not only help advance basic research but also help reduce the use of animals in biomedical science (10). 3D organoid methods are also very important for the study of diseases in conditions when it is difficult to obtain patient tissues. Therefore, developing methods to rapidly and accurately process organoid image data become a top priority. Accelerating the analysis of organoid image data is also extremely important for the development of biological experiments. A bladder cancer organoid derived from a patient is an *in vitro* model of their bladder tumour, and it can be used as a model to rapidly select for the most appropriate therapeutic strategies for each individual patient (6, 11, 12). The great advantage of the bladder cancer organoid is that this model is an accurate mimic of the bladder cancer patient’s tumour (7). At present, the role of the drug sensitivity prediction model in the precision treatment of bladder cancer has gradually become prominent. This model is able to determine the most sensitive drugs for patients *in vitro* and evaluate their therapeutic effects. The introduction of organoid models into the diagnosis and treatment of bladder cancer not only predicts resistance to anti-cancer drugs but also identifies effective cancer therapy for individual patients (13, 14).

Deep learning is a machine learning method based on artificial neural networks with representation learning. By using rapid and continuous automatic analysis of pathological images, the deep learning method could dramatically improve the accuracy rate and efficiency of drug screening and evaluation. Despite the rapid development of deep learning applications in pathological images, such as retinal segmentation (15), and pulmonary nodule segmentation (16), development of a deep learning segmentation method suitable for cancer organoids, especially for evaluation of bladder cancer organoids and drug screening, requires more efforts and is less studied.

**Figure 1:**
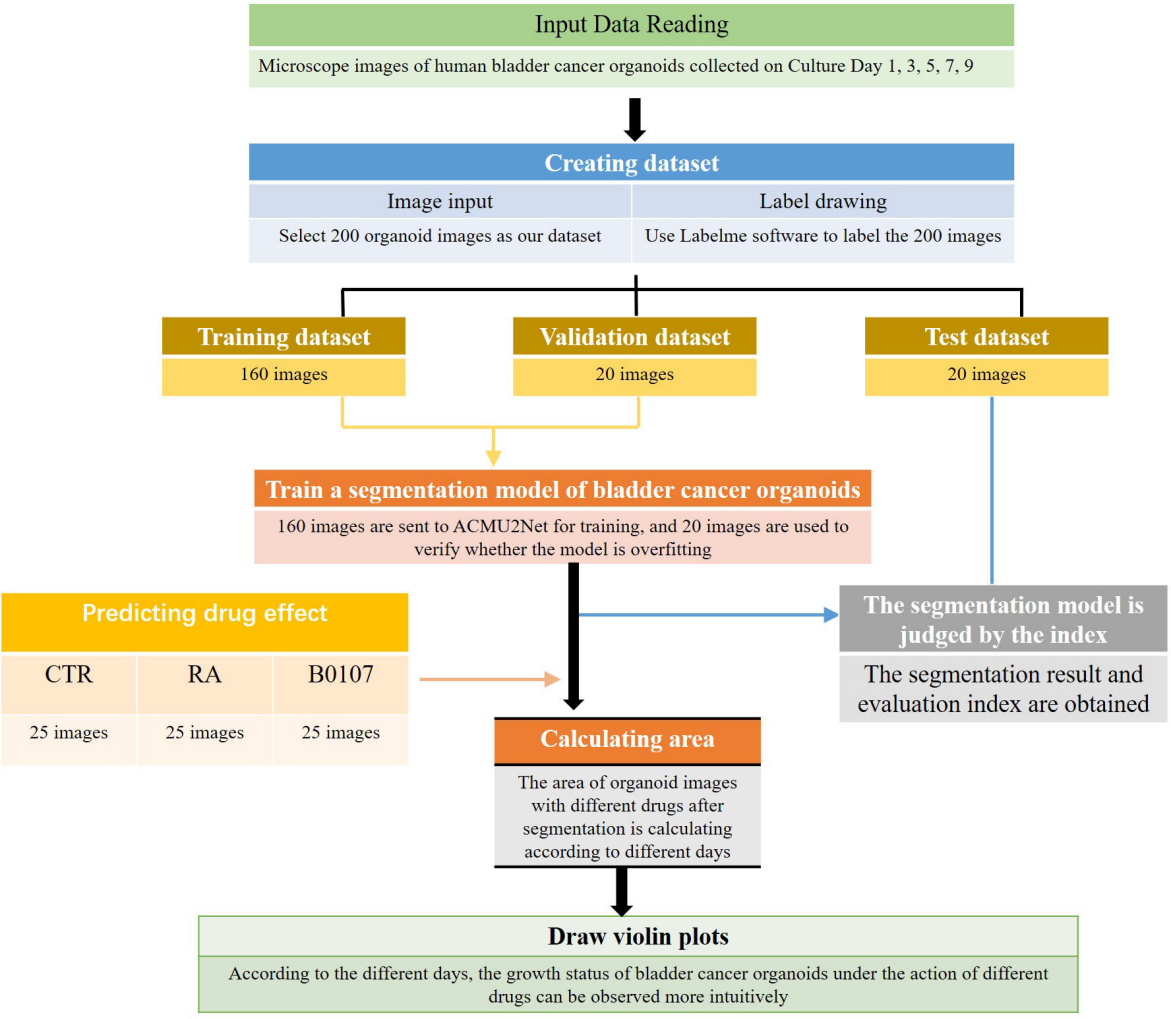
Workflow of using ACU2Net model for drug screening for bladder cancer organoids.

This paper proposes a new Attention and Cross U2Net (ACU2Net) model for bladder cancer organoid segmentation in images. We used an image segmentation neural network in deep learning for bladder cancer organoid segmentation. First, 200 images SW780 and T24 were selected as our data set. Since image segmentation is mainly done by supervised training, our data set has no labels. Therefore, we used Labelme to label the obtained images with masks and outline each organoid. Then, combined with the data characteristics that our organoid background region is more than the target region, we optimized the original U2Net network model and selected a better loss function to improve the segmentation accuracy of the model. After segmentation, the trained model was further used to quickly calculate the area of bladder cancer organoids treated with or without different drugs on different culture days. To quantify the growth of organoids, violin plots were generated permitting the easy visualization of the effect of drug treatment on different days. The workflow for this process is shown in **Figure 1**.

Our innovations are that this is the first time the image segmentation network has been applied in the application of bladder cancer organoid segmentation. Our model also added a grouping and merging module to the original U2Net (17) jump connection. This combination enriched the characteristic information of bladder cancer organoids, especially the semantic and spatial characteristics. An improved attention mechanism (18) was introduced to make the feature extraction network pay more attention to key features in the feature layer and spatial region of bladder cancer organoids while ignoring unnecessary features in redundant regions. After segmentation, the computer is used to automatically determine the growth of organoids treated with different drugs or different drugs at different days, and then generate violin diagrams for faster and easier visualization for drug screening. The ACU2Net model was developed and tested on 3D bladder cancer organoids for drug screening and evaluation. Our results show that the ACU2Net model is faster, as traditional manual bladder organoid drug screening takes 2-3 days, while the deep learning algorithm only takes about 1 hour and is more accurate. In summary, our method can provide more accurate segmentation results for organoid images and greatly improve the efficiency of drug screening evaluation.

## 2 Related work

### 2.1 Clinical trial simulation

Currently, drug screening strategies have been developed based both on experimental and virtual methods. Experimental screening refers to screening in the laboratory. Clinical trial simulation, a virtual screening method, has recently emerged as an interdisciplinary subject and attracted widespread attention in the pharmaceutical industry (19–21). Statistics show that during the stages of new drug development, 60% of drugs fail due to poor drug metabolism or high toxicity. The emergence of clinical trial simulation can guide experimental design in drug research, by conducting computer simulations on key hypotheses before bench experiments, and can potentially obtain drug effect information. Therefore, clinical trial simulation can reduce research investment in clinical trials and improve research efficiency and success rates (22).

Since the discovery of new drug targets and action mechanisms has become more complex, drug screening nowadays requires a huge investment of time and money. To solve this problem, many pharmaceutical companies and drug research institutions have applied artificial intelligence, such as deep learning methods (23), to improve screening efficiency and to mine for new findings from existing data. For example, DOCK Blaster (24), was developed by the Shoichet Laboratory for molecular docking. It screens in a specific database with a given receptor structure to search for potential active small molecules. However, the parameter settings for this method are troublesome and often influence the accuracy and reliability of the results. Iscreen (25), developed by the Taiwan YC Laboratory, can conduct virtual screening of traditional Chinese medicines online, but only on a small scale, with low diversity and a long screening time. DeepScreen (26) uses convolutional neural networks to obtain single-cell images based on flow-cytometry cells. Compared with standard experimental methods, DeepScreen can significantly reduce the detecting time, from a few days to 2-6 hours, effectively improving the detecting efficiency.

### 2.2 Image segmentation network

The concept of deep learning originated from the research of artificial neural networks. The structure of deep learning is a multi-layer perceptron structure with multiple hidden layers. It combines low-level features and then forms more abstract high-level features to represent attribute categories or features. Deep learning theory includes many different deep neural network models (27), such as classic deep neural network (DNN), convolutional neural network (CNN), deep Boltzmann machine (DBM), and recurrent neural network (RNN). Networks with different structures are suitable for processing different data types. For example, CNN is suitable for image processing, and RNN is suitable for speech recognition. At the same time, some different variants of these network models will be produced by combining them with different algorithms.

Deep learning has significantly progressed in image classification (28), image segmentation (29), and object detection (30). Deep learning has also been gradually applied in the field of biomedical image segmentation in natural image segmentation. For example, one group (31) used a fully convolutional network to solve the task of cell image segmentation. The network can classify each pixel and achieve end-to-end training, but the pooling operation causes a slight loss of adequate information, which is less effective in fine-grained segmentation. Another group (32) proposed U-Net, to compensate for information loss through deconvolution layers and feature stitching. It has a simple structure, few parameters, and strong plasticity. As a result, it is one of the most basic and effective models currently applied to cell image segmentation. However, the network is not practical for edge contour segmentation, because there are many cell breakage problems and the depth of the network that can be constructed is limited due to vanishing gradients problems. To improve the U-Net model, some deep learning networks have been proposed. For instance, UNet++ (33) connects encoders and decoders using dense skip connections between different layers. In addition, FU-Net (34) uses a dynamically weighted cross-entropy loss to improve U-Net. Quan et al. (35) combined the features extracted by the segmentation network to build a more profound network architecture to achieve more accurate cell segmentation. Moreover, attention mechanisms have recently been used in image segmentation to improve the segmentation effect. For example, Fu et al. (36) proposed a dual attention network attention mechanism that captures contextual information dependencies through a self-attention mechanism and adaptively integrates local features and global information. However, efficiency was only mildly improved through this model, as well, in this model there are too many parameters and it takes too much run time.

## 3 Methods

**Figure 2:**
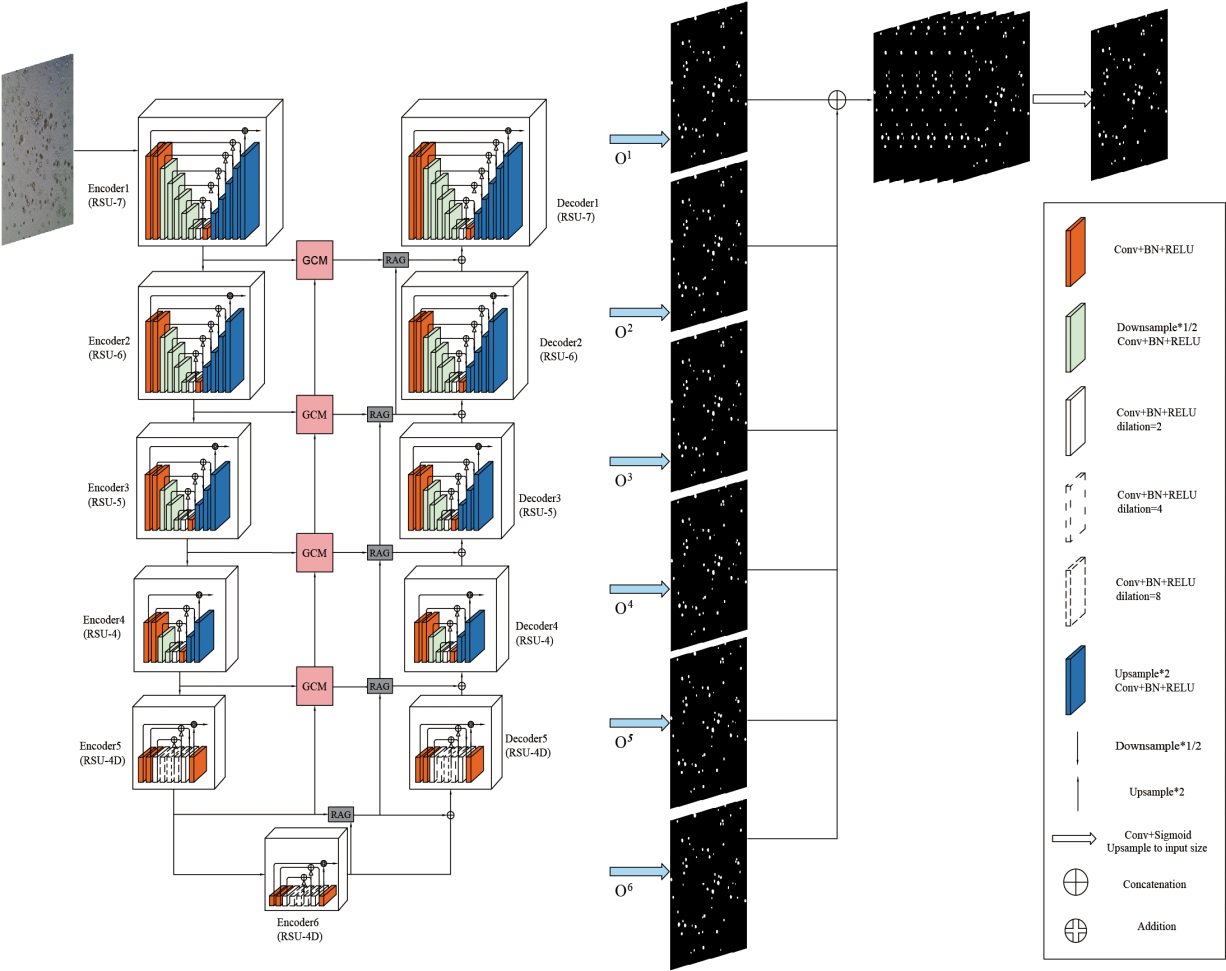
Overall architecture of ACU2Net model.

Given the characteristics of bladder cancer organoids in microscope images, this paper introduces Grouping Cross Merge (GCM) in the skip connection of the U2Net network on the basis of U2Net and Attention U-Net (37) and combines GCM with the encoder and decoder of the U2Net network. The shallow and deep features are cross-merged by the GCM module. In addition, to further enhance the correlation between features, a feature transfer channel is added between each GCM module, so that the features of the entire U2Net network are serially passed to the last GCM module and output. Next, it is passed through the Residual Attention Gate (RAG), which enhances the model’s ability to extract the features of the region of interest. Finally, bladder cancer organoids and backgrounds are classified through a 1×1 convolutional layer. The proposed model is shown in **Figure 2**.

### 3.1 Residual U module

**Figure 3:**
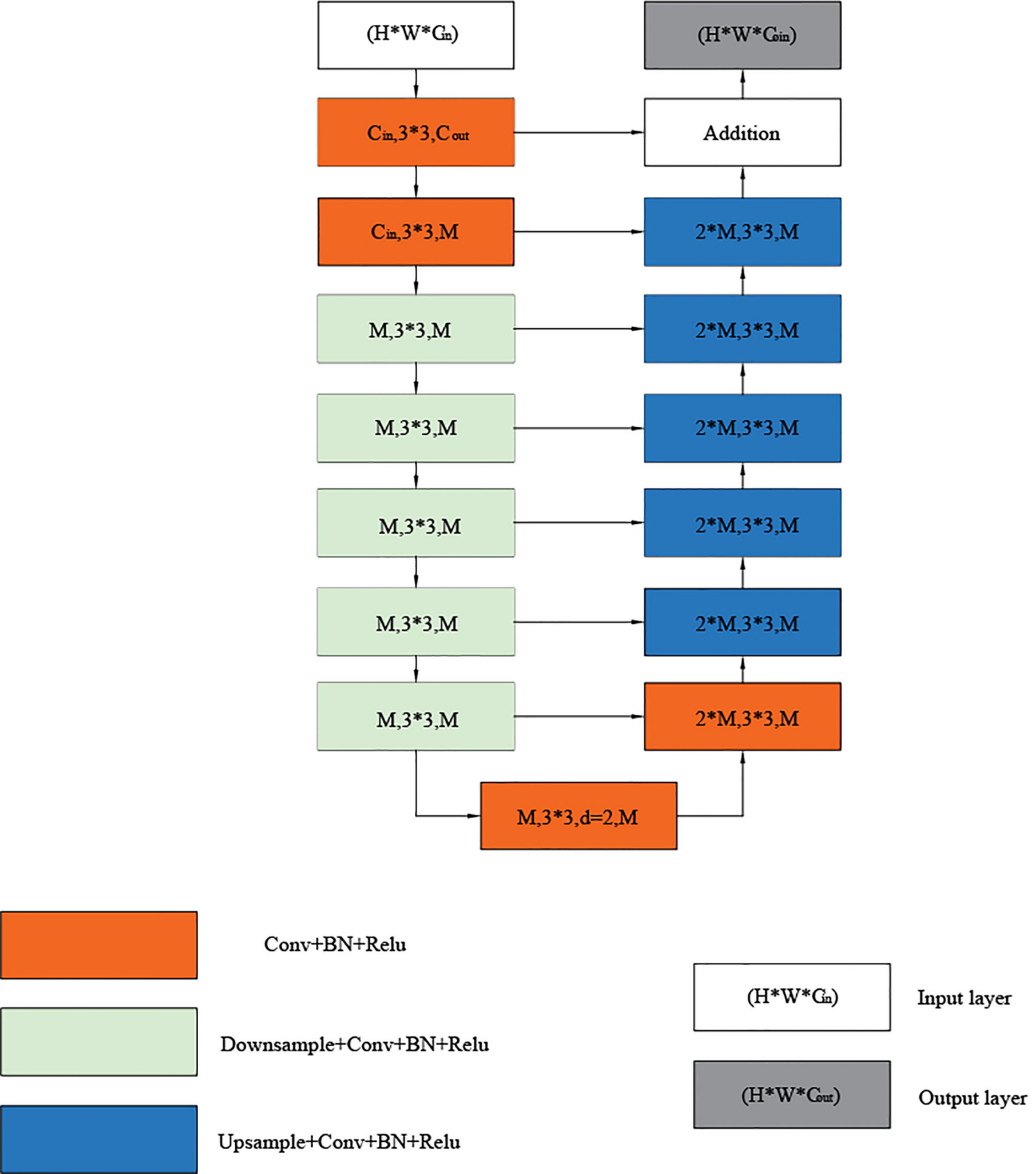
Overall architecture of RSU method.

The residual U-module consists of three parts: an input layer that collects local features and transforms channels, a U-shaped structure that extracts and encodes multi-scale context information, and an output layer that fuses the input and intermediate layers. First, the convolutional layer, which is a standard for local feature extraction, converts the input feature *x*(*H*×*W*×*C_in_*) map into an intermediate *F_1_* (*x*) map with *C_out_* channels. Next, a U-shaped symmetric encoder-decoder structure with a height of L, which takes the intermediate feature map *U* (*F_1_* (*x*)) as input, learns to extract and encode multi-scale context information, and finally fuses the residual connections of local features and multi-scale features by summing. The U-shaped symmetric codec structure representation is shown in **Figure 3**, where L is the number of layers in the encoder, *C_in_* and *C_out_* represents the number of input and output channels, and M represents the number of channels in the RSU middle layer. The more significant L is, we can get a deeper residual U-shaped block (RSU), more pooling operations, more diversity of the receptive field, and richer local and global features. Therefore, we can configure the level parameter L to extract multi-scale features from input feature maps with arbitrary spatial resolution. In this paper we selected L=7. When using larger L, the sampling of the feature graph will lead to the loss of useful context, resulting in higher computing and memory costs. Using a smaller L less local semantic information can be captured. In contrast, L=7 can capture global semantic information and local semantic information and consume fewer computing resources and computing costs.

### 3.2 The overall structure of the U2Net module

The overall structure of the U2Net network includes 6 encoders and 5 decoders, as well as a saliency map fusion module connected to the decoder. The configured RSU-L fills each stage, and the level parameter L is configured according to the spatial resolution of the input feature map. As can be seen from **Figure 2**, the left part of the network is the down-sampling process. The first 4 stages fill Encoder1, Encoder2, Encoder3, and Encoder4 with RSU, whose level parameters L are 7, 6, 5, and 4, respectively. For feature maps with larger resolutions, we use a more significant L to obtain information at a larger scale, and the size of the feature maps in each stage is halved layer by layer and restored to the original size. The feature maps in Encoder5 and Encoder6 are of relatively low resolution, and further down-sampling of these feature maps will result in the loss of adequate contextual feature information. Therefore, RSU-4D is used in both stages, where “D” indicates that RSU is a dilated version in which we replace the pooling and up-sampling operations with dilated convolutions, which means that all intermediate feature maps of RSU-4D have the same resolution as its input feature maps. The last two stages are filled by RSU configured with dilated convolution, and the size of the feature graph within the stage remains unchanged. In the encoding stage, the RSUs are connected by a 2×2 max pooling, and the feature map size becomes 1/32 of the original. The right side of the network is a decoding module composed of 5 decoders, which is an up-sampling process. The RSU configuration in each stage is the same as the left symmetrical position, so the decoder has a similar structure to its symmetric encoder. In Decoder5, we also use an extended version of the residual U-block RSU-4D, similar to that used in encoder-level Encoder5 and Encoder6. The input for each decoder level is a decoder output from its previous level and a symmetric encoder output. In addition, the decoding part changes the size of the reduced feature map to the original size.

The last part is the feature fusion module, which generates segmentation probability maps. U2Net first generates six output feature maps *O^6^*, *O^5^*, *O^4^*, *O^3^*, *O^2^*, and *O^1^* from Encoder6, Decoder5, Decoder4, Decoder3, Decoder2, and Decoder1 through a 3×3 convolutional layer and a sigmoid function. Then, it up-samples the side output feature map to the input image size, and the six output feature maps are spliced to generate the final segmentation map. The network structure parameters of U2Net are shown in **Table 1**.

**Table 1:** Network parameters of U2Net.

	Block	input size (C × H × W)	output size (C × H × W)	Note
Decoder and Encoder Part	Encoder1	3×256×256	3×256×256	Max Pooling
	Encoder2	64×128×128	128×128×128	Max Pooling
	Encoder3	128×64×64	256×64×64	Max Pooling
	Encoder4	256×32×32	512×32×32	Max Pooling
	Encoder5	512×16×16	512×16×16	Max Pooling
	Encoder6	512×8×8	512×8×8	Dilated Conv
	Decoder5	512×16×16	512×16×16	Dilated Conv
	Decoder4	512×32×32	256×32×32	Up-sampling
	Decoder3	256×64×64	128×64×64	Up-sampling
	Decoder2	128×128×128	64×128×128	Up-sampling
	Decoder1	64×256×256	32×256×256	Up-sampling
Fusion Part of Feature Figure	*O^6^*	512×8×8	1×8×8	Conv+Sigmiod
	*O^5^*	512×16×16	1×16×16	Conv+Sigmiod
	*O^4^*	256×32×32	1×32×32	Conv+Sigmiod
	*O^3^*	128×64×64	1×64×64	Conv+Sigmiod
	*O^2^*	64×128×128	1×128×128	Conv+Sigmiod
	*O^1^*	32×256×256	1×256×256	Conv+Sigmiod

### 3.3 Grouping cross fusion module

**Figure 4:**
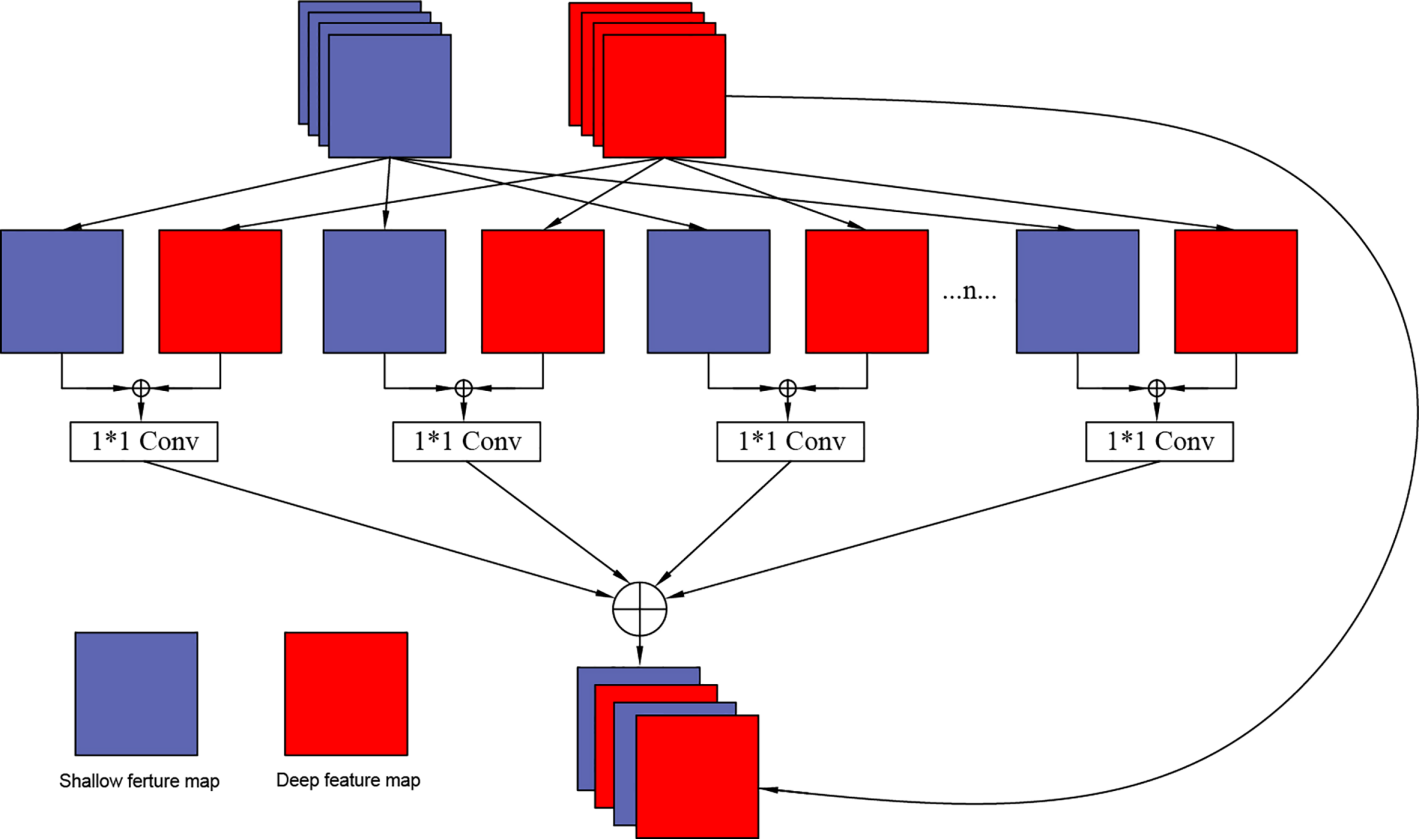
Our proposed grouping cross merge (GCM) module.

In this design, to further improve the correlation of feature information, the Grouping Cross Merge (GCM) is applied in the U2Net network, which fuses low-level morphological features and high-level semantic features to enrich the feature information. The network structure of the packet cross-fusion module designed in this paper is shown in **Figure 4**.

There are two input feature maps for the GCM module: the “mirror” feature maps for the shallow network and the deep network feature maps for the U2Net network. Each feature map is divided into n groups. Because the channel number of the shallow network is relatively small, and the channel number of the deep network is relatively large, we set the values of n as 4, 6, and 8 according to the different layers of the network, shallow layer, middle layer, and deep layer, respectively. Based on the granularity that needs to be subdivided, the two groups of features are merged using the CONCAT layer. After merging, the feature maps are integrated using a 1 x 1 convolutional layer, and the number of channels of the feature map is halved. At this stage the feature cross-processing of each group is completed. Next, the CONCAT layer is used to combine again to complete the cross- of the two feature maps. Since many convolution calculations are involved in the entire module, to prevent problems such as vanishing gradient, the input feature maps in the deep network are mapped to the cross-merge modules to construct the residual channels of the overall module.

### 3.4 Residual attention gate

**Figure 5:**
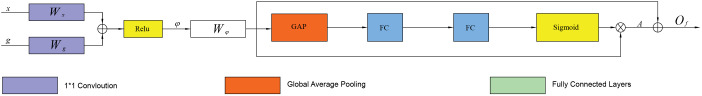
The residual attention gate.

In the Residual Attention Gate (RAG), the feature map of the same layer in the contraction path and the feature map of the previous layer in the expansion path achieve multi-scale feature fusion through convolution, addition, and activation operations. Then, 1×1 convolution filtering is performed on the fused features, and global average pooling (GAP) is used to extract global features to reduce dimensionality (38). Next, two fully connected layers (FC) are combined with the sigmoid function to generate an organoid-oriented weight matrix, which is then multiplied by the feature fusion graph to adjust the feature weights (39). Since the weight matrix continuously assigns weights to the target area, the value of the background area in the output feature map will be smaller, and the value of the target area will be more significant. Finally, the feature fusion map is residually connected with the adjusted weight feature map to obtain the final output features. The overall structure of the RAG is shown in **Figure 5**.

First, the feature maps ***x*** ∈ ***R*** ^***C***_*x*_× ***W*** × ***H***^ and ***g*** ∈ ***R*** ^***C***_***g***_× ***W***/**2**× ***H***/**2**^ are converted from different scale layers into a unified size and mapped to the feature spaces of *a*, *b*, respectively. Then, a 1 x 1 convolution is performed, and a bit-by-bit summation is carried out. Finally, the fused feature map is obtained by activating the Relu function. The feature map can be defined as ***φ***:


(1) $$\varphi =Relu\left(a\left(x\right)+b\left(upsampling\left(g\right)\right)\right)\in R^{C\times H\times W}$$

In the formula, *a*(*x*)*=W_x_x*, *b*(*g*)*=W_g_g*, then the feature *φ* is sent to the convolutional layer to filter the multi-scale features to generate another feature space d, where ***d*** (***φ***) **=*W_φ_φ***. *W_x,_ W_g_*, and *W_φ_* represent the 1×1 convolution operation. Next, the GAP layer is applied for dimensionality reduction and global feature extraction to convert it into a one-dimensional vector ***a*** (***d*** (***φ***)) ϵ ***R*** *^1×C^*. Afterwards, two fully connected layers, *f_c1×c/r_* and *f_c1×c_* are used to model the correlation between channels (the first layer has c/r channels, the second layer has c channels, and r is the reduction ratio), and outputs the same number of weights as the input features. This operation has more nonlinearity, so that it can better fit the complex correlation between channels, and significantly reduce the number of parameters and computation. Then a Sigmoid function is applied to obtain weights normalized between 0 and 1 to generate a channel attention map *A*:


(2) $$A=sigmoid\left(f_{c1\times c}\left(Relu\left(f_{c1\times c/r}\left(b\left(d\left(\varphi \right)\right)\right)\right)\right)\right))$$

For each channel ***A_k,_ k*** ϵ {1,…, *c*} in the attention map ***A*** ϵ ***R*** *^1×C^*, the weight of the feature map ***d*** (***φ***) ϵ **R^C^** ^×^ **^H^** ^×^ **^W^** is adjusted. The above calculation retains the useful semantic and detail features of different layers, and the complex background is suppressed as much as possible. Finally, we perform the residual operation, adding pixels with the fusion feature graph to obtain the final output feature *o_f_.*


(3) $$o_{f}=\sum_{k=1}^{c}A_{k}d\left(\varphi_{k}\right)+d\left(\varphi \right)$$

### 3.5 Loss function

The segmentation of bladder cancer organoids is a pixel-level binary classification problem, and we adopt a binary cross-entropy function (40) as part of the loss function. Its mathematical expression is:


(4) $$L_{BCE}=-\frac{1}{n}\sum^{}(y_{n}\times lnx_{n}+\left(1-y_{n}\right)\times ln\left(1-x_{n}\right))$$

Among them, *y_n_* = {0,1} represents the true value of the dataset, and *x_n_* ={0,1} represents the prediction result.

In medical image analysis, the Dice coefficient is often used as a criterion for judging segmentation results. The Dice loss function proposed based on the Dice coefficient can show whether the prediction results and the data manually labelled by experts show a consistent distribution in the overall performance (41). At the same time, the Dice loss function focuses on mining the foreground area’s information during the network training process. We take the Dice loss function as another part of the loss function, whose mathematical expression is as follows:


(5) $$L_{Dice}=1-\frac{2\times \left|Pred\cap^{}Label\right|}{\left|Pred\cap^{}Label\right|}$$

Where *Pred* denotes the predicted image of the network and *Label* denotes the standard ground truth image.

The overall loss function of the ACU2Net network combines the binary cross-entropy loss function and the Dice loss function with the following mathematical expressions.


(6) $$L_{loss}=\;L_{BCE}+L_{Dice}$$

## 4 Experimental results

### 4.1 Dataset and experimental setup

**Figure 6:**
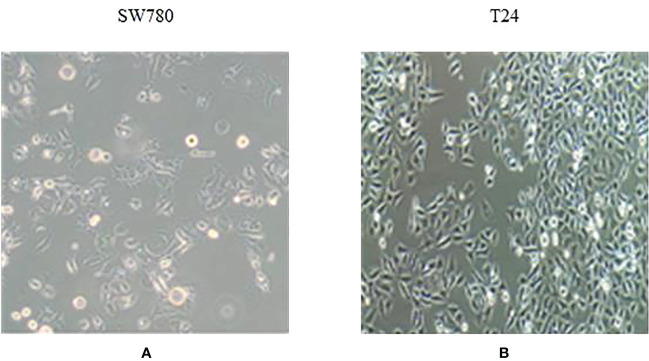
Bladder cancer cell line organoid model. **(A)** SW780 bladder cancer cell line. **(B)** T24 bladder cancer cell line.

**Figure 7:**
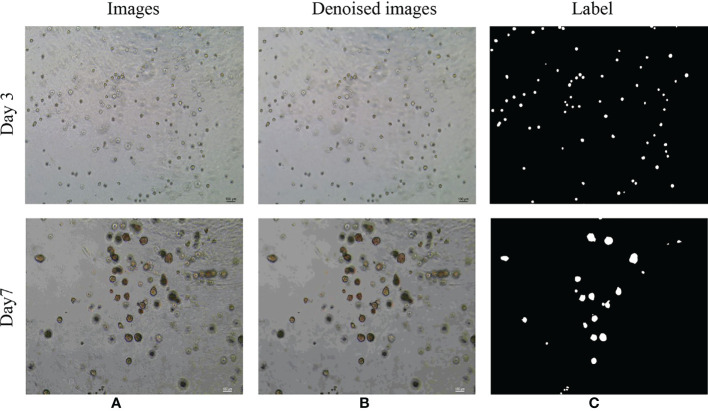
Examples of the original microscope image and corresponding denoised images of bladder cancer organoids. **(A)** Microscope images of bladder cancer organoids on culture day 3 and day 7 **(B)** Denoised images based on the microscope images of bladder cancer organoids. **(C)** The labels generated from denoised images of bladder cancer organoids.

The dataset used in this study is the image of bladder cancer organoids provided by the Life Science Center of Yunnan University. The data set included 200 images from bladder cancer organoids. The pixel size is 2592 × 1944. SW780 and T24 bladder cancer cell lines were used to establish the organoid model (**Figure 6**). SW780 cells came from low malignancies female patient representing bladder cancer clinical stage I (T1), while T24 cells came from high malignancies female patient representing bladder cancer clinical stage 3 or above. In brief bladder cancer cells were digested by 0.125% or 0.25% trypsin EDTA (Viva Cell, C3530, China) for 3-5 minutes, after 1200 rpm centrifuge, bladder cancer cells were counted and diluted into 250 cells/μl. The Cultrex Reduced Growth Factor Basement Membrane Extract, Type 2, PathClear (BME, R&D, 3533-010-02, USA) was prepared on ice, and then added 4 μl resuspended bladder cancer cells into 40uL BME on ice and mixed gently, finally loaded into a well of 24 wells plate in 37C for 10 minutes. Once BME solidified, 600uL complete DMEM cell culture medium was added into 24 wells plate, and medium were replaced in every 3 days. Organoid images were collected by Microscope DM2000 (Leica, German) at day 1, 3, 5 and 7. The complete culture medium for SW780 bladder cancer organoids was composed of RPMI Medium 1640 basic (Gibco, C118755008T, USA), 10% Fetal Bovine Serum(FBS, Gibco, 10099141C, USA), 1% Penicillin-Streptomycin (PS, Millipore, TMS-AB2-C, USA), 1% HEPES (Viva Cell, C3544-0100, China), while the medium for T24 cell organoids contained McCoy’s 5A Medium (Viva cell, c3020-0500, China), 10% Fetal Bovine Serum(FBS, Gibco, 10099141C, USA), 1% Penicillin-Streptomycin (PS, Millipore, TMS-AB2-C,USA).Since the drug was diluted by DMSO(sigma, D2650, China), the medium of CTR group contained the same proportion of DMSO as the control, while the medium of RA(MedChemExpress, HY-14649, China) and B0107 (MedChemExpress, HY-B0107, China) groups contained RA and B0107. Because the images were collected at different times and from different environments, there exists uneven staining and illumination in the images, therefore it is necessary to denoise them. For this purpose, we chose the non-local average denoising algorithm (42) for processing. Each image is a ground truth image marked by experts at the pixel level. An example of the images in the dataset and its pre-processing are shown in **Figure 7**.

In order to make full use of the limited dataset, the denoised dataset was expanded by random flipping, cropping, and scaling. Therefore, the 200 images of bladder cancer organoid were expanded to 6400 bladder cancer organoid images with a size of 256×256 pixels. Moreover, the dataset was randomly divided into a training set, a validation set, and a test set in a ratio of 8:1:1, respectively. The experimental environment configuration required for experiments is shown in **Table 2**.

**Table 2:** Experimental environment configuration.

Configuration	Parameter
OS	Ubuntu20.04
CPU	i9 7900
GPU	Geforce GTX 3090 GPU×2
CUDA	CUDA 11.1
Environment	Python 3.8
Frame	Pytorch 1.7.1

A total of 1000 epochs were trained, and the gradient descent was optimized using the SGD (Stochastic Gradient Descent) algorithm (43). In addition, loss functions use Dice Loss and BCE Loss, and the neuron inactivation probability was set to 0.5 (44), which is used to further reduce the overfitting phenomenon.

### 4.2 The evaluation index

The purpose of bladder cancer organoid segmentation is to divide the pixels in the organoid image into organoid pixels and background pixels. There are four possible segmentation results: True Positive (TP), which indicates that the pixels in the organoid images marked by experts are classified correctly as organoids; False Negative (FN), indicating that the organoid pixels in the images marked by experts are classified incorrectly as background; True Negative (TN), indicating that the background pixels in the organoid images marked by experts are classified correctly as background; False Positive (FP), indicating that the background pixels in the images marked by experts are classified incorrectly as organoids. In order to evaluate the segmentation results, four evaluation indicators of accuracy (Acc, accuracy), sensitivity (Se, sensitivity), specificity (Sp, specificity), and F1–Score are used to evaluate the segmentation effect objectively. The definitions of evaluation indicators are shown in **Table 3**.

**Table 3:** The evaluation index.

Evaluation indicators	Formula
*Acc*	$Acc=\frac{TP+TN}{TP+TN+FP+FN}$
*Se*	$Se=\frac{TP}{TP+FN}$
*Sp*	$Sp=\frac{TN}{TN+FP}$
*F1–Score*	$F1-Score=\frac{2\times TP}{2\times TP+FP+FN}$

### 4.3 Results analysis of different segmentation models

**Figure 8:**
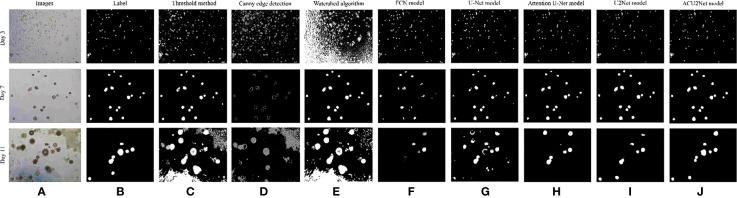
Comparison of results of different segmentation methods. **(A)** Image of bladder cancer organoids. **(B)** The bladder cancer organoids label. **(C)** Threshold segmentation method. **(D)** Canny edge detection. **(E)** Watershed algorithm. **(F)** FCN model. **(G)** U-Net model. **(H)** Attention model. **(I)** U2Net model. **(J)** ACU2Net model.

This paper first tested the traditional segmentation algorithm to segment organoids. Three traditional segmentation algorithms were selected, which are the threshold segmentation algorithm (45), the canny edge detection algorithm (46), and the watershed algorithm (47). The results are shown in **Figure 8**. We can see from **Figures 8C–E** that the three traditional segmentation algorithms have a good ability for organoid segmentation in a simple background, but it is quite different from tags for organoid segmentation in complex backgrounds. Next, we compared four algorithms of classical deep learning neural networks which are the FCN model, the U-Net model, the Attention U-Net model, the U2Net model, and our proposed ACU2Net model. These results are shown in **Figures 8F–J**. The segmentation results of the FCN model are not precise enough and incomplete, and there are a large number of unrecognized organoids. The segmentation results of the U-Net model have improved with obvious edges, but there are still problems of mis-segmentation in images with complex backgrounds. The Attention U-Net model has better segmentation results, and is less affected by background interference, but its segmentation outline is slightly rough. The U2Net model has precise segmentation edges and achieves better results in images with a large number of organoids, but in images with complex backgrounds, some organoids are often mis-segmented. In images with complex backgrounds and many organoids, the proposed ACU2Net network model can maintain a complete structure, and it can usually segment more detailed information in regions with weaker edges.

Although it is more intuitive to observe with the naked eye, subjective factors may still be present, so it is still necessary to quantitatively evaluate the segmentation results. These results are shown in **Table 4**. The performance of our ACU2Net model is better than other models on the four evaluation indicators of Acc, Se, Sp, and F1–Score, which proves the rationality and effectiveness of the model. In addition, it can segment more organoid information in complex background regions, and has strong robustness. Due to the insufficient recovery of detailed information during the up-sampling process, the segmentation effect of the FCN model is slightly worse. By introducing deconvolution and feature layer connections, the U-Net model makes up for the lost detail information to a certain extent, and the effect is greatly improved. Furthermore, the Attention U-Net model uses the attention module to replace the traditional method of direct connection in the U-Net network, which enhances the effectiveness and selectivity of the connection in the effective connection of primary features and advanced features, and all indicators are significantly improved. The U2Net model utilizes a nested U-shaped network that integrates features of receptive fields of different sizes to segment most organoids. Compared with the U2Net model, the proposed model improved in various indicators, indicating that the proposed model can segment more organoids in complex areas and areas with blurred background edges, so it is more adaptable to interference factors such as contrast and noise, and the segmentation effect is better than other models.

**Table 4:** Test results of different segmentation algorithms. Bold represents the best of each category.

Model	Acc(%)	Se(%)	Sp(%)	*F1–Score*(%)
FCN	97.83	83.47	81.40	82.42
U-Net	98.82	89.35	83.75	86.45
Attention U-Net	98.92	91.75	82.63	86.96
U2Net	98.97	92.64	84.97	88.64
ACU2Net	**99.23**	**94.81**	**88.50**	**91.54**

### 4.4 The influence of each module on the overall model

In order to verify the effectiveness of the RAG and GCM modules introduced in this paper, the network was adjusted as follows (1): the original U2Net network, is represented by Net1 (2); the U2Net network, which is combined with RAG module, is denoted by Net2; (3) the U2Net network, which is combined with GCM module, is represented by Net3; (4) The U2Net network, which is combined with RAG and GCM modules, is denoted by Net4. The influence of different modules on the model is shown in **Table 5**.

**Table 5:** Influence of each module on the whole model. Bold represents the best of each category.

	U2Net	RAG	GCM	Acc (%)	Se (%)	Sp (%)	F1–Score (%)
Net1	+			98.97	92.64	84.97	88.64
Net2	+	+		99.12	**95.43**	86.63	90.82
Net3	+		+	99.01	94.19	85.74	89.77
Net4	+	+	+	**99.23**	94.81	**88.50**	**91.54**

**Figure 9:**
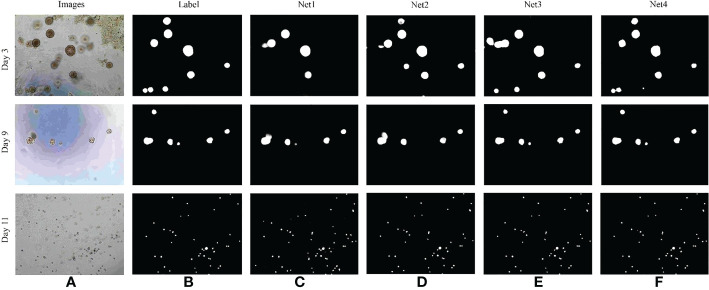
Comparison of results of different segmentation methods. **(A)** Image of bladder cancer organoids. **(B)** The bladder cancer organoids label. **(C)** Net1 model. **(D)** Net2 model. **(E)** Net3 model. **(F)** Net4 model.

As can be seen from **Table 4**, the F1–score of the original U2Net network segmentation is 88.64. When the RAG module is added to the jump connection of the original U2Net network, the F1–Score of the network is 2.18% higher than that of the U2Net network, indicating that RAG attention can extract more detailed feature information about bladder cancer organoids and reduce the influence of a lot of background redundancy. It helps to combine high-level semantic information with low-level fine-grained surface information to improve the recognition ability. Compared with the U2Net network, the F1–Score of the GCM module is improved by 1.13%, indicating that the GCM module can maximize the feature transmission of significant regions of bladder cancer organoids, and the details of small targets would not be lost with the deepening of the feature extraction process. Finally, when the two modules are added to the whole network simultaneously, the F1–Score is improved by 2.91%, indicating that the proposed algorithm has a good segmentation effect on the image of bladder cancer organoids with complex structures, which fully proves the effectiveness of the proposed algorithm. The test results of each module are shown in **Figure 9**.

The reasons for this improvement are: Based on the structure of U2Net, the channel attention network is inserted into the U2Net skip connection part, which improves the algorithm’s ability to learn the characteristics of bladder cancer organoids and the ability to process detailed information. As well,

ACU2Net can capture the spatial relationship with the surrounding pixels by applying high-resolution images in different layers of networks, but the ability to capture detailed features is poor. In addition, it can focus on the geometric details of the image by utilizing low-resolution images to obtain the local receptive field of the network, which is suitable for high-precision segmentation tasks, but the ability to represent images needs to be strengthened. Therefore, the different receptive fields obtained by the GCM module can further improve the image representation ability, to solve the problem of various sizes of bladder cancer organoids.

### 4.5 Drug screening evaluation

**Figure 10:**
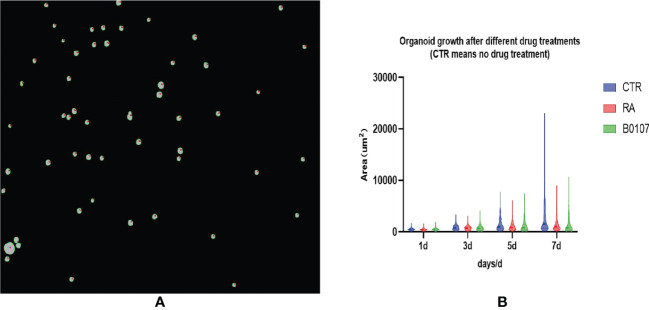
Drug screening evaluation based on ACU2Net model. **(A)** A micro image of organoids treated with drugs. **(B)** Using ACU2Net methods, the growth results of organoids after treatment of (retinoic acid) RA and B0107.

After training the model to identify bladder cancer organoids, segmentation is completed, and drug screening is required to select candidates for the treatment of bladder cancer for clinical trials. First, the segments as shown in **Figure 10A** need to be automatically computed to build a violin diagram, which can be drawn by calculating the organoid area after treatment with different drugs on different days. **Figure 10B** reflects the growth of bladder cancer organoids from 1 to 7 days in three environments, where the CTR group represents no drug treatment, and RA and B0107 are the abbreviations for the names of the two drugs used to treat bladder cancer, respectively. To observe the growth of organoids more intuitively, a quartile distribution map is added to the violin plot, and the dotted line in the middle is the median of this data group. As can be seen from the figure, as days 1-3 are the initial growth stage of bladder cancer organoids, there is no significant difference between drug-treated and non-drug-treated organoids. The growth differences gradually appear after day 5, with the organoids in the CTR group reaching a peak. The median area of CTR is higher than those of RA and B0107, indicating that these two drugs have a specific inhibitory effect on the growth of bladder cancer organoids and can be put into clinical development. To verify the reliability of the experiment, we invited professionals in related fields to perform manual verification. These experts confirmed that the results of this paper showed the same regularity as the manual statistical results. While manual data processing time was 6 hours, the deep learning method used in this paper can screen and evaluate bladder cancer organoids in about 10 minutes. Therefore, the proposed method can promote the throughput for the testing of anti-cancer drugs for bladder cancer.

## 5 Conclusions

In this paper, an imaging segmentation method for bladder cancer organoids is proposed by using the U2Net basic framework combined with residual attention gate and grouping cross fusion module. The method employs GCM, the grouped cross merge module, to obtain objects of different sizes at the skip connection of the model, which improves the feature representation of segmentation. Through integrating the improved attention mechanism, RAG, the semantic information of the feature is enhanced for finer segmentation, and the F1-Score indicator has reached 91.54% in this case. In addition, the application of this new method in evaluating the growth status of organoids on different culture days, with or without the treatment of drugs, found it could provide accurate screening results with higher efficiency for drug screening. This evidence shows that the proposed novel algorithm has a greater improvement in the imaging segmentation of bladder cancer organoids, especially in drug screening evaluation using bladder cancer organoids, than the existing algorithms. However, there are also some limitations to this method. Due to the complex background of the 2D images of bladder cancer organoids on Day 7, the adhesion of the organoids and the existence of aliasing, noise, and mixing effects of sampling points cannot be solved by image segmentation alone. In this case, the organoid volume for drug screening should also be considered. Therefore, the 3D reconstruction of bladder cancer organoids cultured for longer than seven days is the main focus of our later research.

## Data availability statement

The original contributions presented in the study are included in the article/supplementary material. Further inquiries can be directed to the corresponding authors.

## Author contributions

All authors listed have made a substantial, direct, and intellectual contribution to the work, and approved it for publication.
